# Financial Burden of Medical Care, Dental Care, and Medicines among Older-Aged Population in Slovenia, Serbia, and Croatia

**DOI:** 10.3390/ijerph19063325

**Published:** 2022-03-11

**Authors:** Katarina Vojvodic, Zorica Terzic-Supic, Jovana Todorovic, Cristina Gagliardi, Milena Santric-Milicevic, Marina Popovic

**Affiliations:** 1 Department for Healthcare Quality Improvement, Institute of Public Health of Belgrade, 11000 Belgrade, Serbia; 2 Faculty of Medicine, Institute of Social Medicine, University of Belgrade, 11000 Belgrade, Serbia; zorica.terzic-supic@mfub.bg.ac.rs (Z.T.-S.); jovana.todorovic@med.bg.ac.rs (J.T.); msantric@med.bg.ac.rs (M.S.-M.); 3 Centre for Socio-Economic Research on Ageing, IRCCS INRCA-National Institute of Health and Science on Ageing, 60124 Ancona, Italy; c.gagliardi@inrca.it; 4 Department of Nuclear Medicine, Institute for Oncology and Radiology of Serbia, 11000 Belgrade, Serbia; marina.popovic1989@gmail.com

**Keywords:** financial burden, healthcare, older-aged, likelihood, medical care, dental care, medicines

## Abstract

The aim was to explore the factors associated with the financial burden (FB) of medical care, dental care, and medicines among older-aged people in Slovenia, Serbia, and Croatia using EU-SILC 2017. The highest frequency of FB of medical care and medicines was in Croatia (50% and 69.1%, respectively) and of dental care in Slovenia (48.5%). The multivariate logistic regression analysis with FB as an outcome variable showed that the FB of medical care was associated with being married (OR: 1.54), reporting not severe (OR: 1.51) and severe limitations in daily activities (OR: 2.05), having higher education (OR: 2.03), and heavy burden of housing costs (OR: 0.51) in Slovenia, with very bad self-perceived health (OR: 5.23), having the slight (OR: 0.69) or heavy (OR: 0.47) burden of housing costs, making ends meet fairly easily or with some difficulty (OR: 3.58) or with difficulty or great difficulty (OR: 6.80) in Serbia, and with being married (OR: 1.43), having heavy burden of housing costs (OR: 0.62), and making ends meet fairly easily or with some difficulty (OR: 2.08) or with difficulty or great difficulty (OR: 2.52) in Croatia. The older-aged have the FB of healthcare, especially the poorest or those with health problems.

## 1. Introduction

Healthcare-related financial burden is defined as the ratio of aggregate family expenditures on healthcare relative to family income [1]. Most often, the financial burden of healthcare is caused by out-of-pocket payments, and people experience it when out-of-pocket payments exceed 40% of the capacity to pay for healthcare [2]. Research on the self-reported financial burden of healthcare provides qualitative information about the need to pay and the consequences of paying out-of-pocket for medical care services [3], and even small payments can cause a financial burden for poor households [4].

Previous studies found that several factors were associated with the financial burden of healthcare, such as household size, presence of family members aged over 65, household members with a chronic disease, income, absence of health insurance, and education level [1,5,6]. The financial burden of healthcare is widely spread among older people in European countries, and the cross-country differences in the distribution of financial burden highlighted the importance of identifying people who are vulnerable [4,7,8]. This was mainly due to medicines, medical products, outpatient and inpatient care, and diagnostic tests [2,4]. Slovenia, Serbia, and Croatia inherited a compulsory health insurance system from the former Yugoslavia, which attempted to provide universal health coverage for the whole population [9,10,11]. A compulsory health insurance scheme financed by contributions is the main source of healthcare financing in these three countries. Health insurance is based on the salaries of the employees, farmers, and self-employed, while retired persons do not pay contributions but are fully covered with health insurance, the same as their family members are [9,10,11]. Emergency healthcare services are fully covered by mandatory health insurance, while the costs of medical services and medicines (from the positive lists) are covered to varying degrees, the rest being covered by co-payments with a defined amount [9,10,11]. The legal acts of these countries define the conditions and the characteristics of those who are eligible for complete or partial reduction of co-payments (poorest socioeconomic status, some diseases or disabilities, but not age) [9,10,11]. Dental care for the older-aged is not fully covered by compulsory health insurance, and patients have to pay co-payments or full or partial price for them [9,10,11]. Long-term care for older-aged, chronically ill, disabled, and other individuals with special needs is provided by the healthcare system and is fully covered by health insurance (in Slovenia) [9] or includes co-payments according to the beneficiary criteria (Serbia) [10], while in Croatia it is mainly organized within the social welfare system financed from the State Budget, with some services provided through the healthcare system (e.g., home visits, transportation) [11]. Across OECD countries, on average, 19% of health spending is paid directly by patients [12]. According to the World Bank, within the general population in 2017, out-of-pocket expenditure as a percentage of health expenditure was 11.0% in Croatia, 12.3% in Slovenia, and 40.6% in Serbia [13]. The most common reasons for out-of-pocket payments in Serbia were for medicines (55.6%), dental services (14.2%), other not specified expenses (11.4%), and private diagnostic services (8.0%) [10]. In Slovenia, these were expenses for dental care (10%) and medicines (4.2%), while in Croatia, for medicines (3.9%) and dental care (2.7%) [14,15]. If out-of-pocket spending represents a high percentage of total health expenditure, it suggests limited financial protection [2]. Previous results showed that 42.5% of primary healthcare users in Serbia have to pay a co-payment for visiting the general practitioner, approximately half had to pay for medicines, and 44.7% for visiting the specialist, and that the most frequent reason for not seeking healthcare was financial obstacles [16,17]. In Croatia, 14% of the population was required to pay co-payments for healthcare services and goods, and the most frequent reason for avoiding healthcare, prescribed medicines, and dental care was costs [18]. Seven percent of the total population in Slovenia reported out-of-pocket expenditures, but the percentage was higher among those with health problems, such as some disability (11%), or among those who reported poor health (15%) [19].

The population is aging all over the world, and it is estimated that in European countries, the share of people over 65 years will grow from 19.1% in 2020 to 30.4% of the total population by 2100, or even more, such as in Slovenia (31.4%,), Serbia (34.4%), and Croatia (35.1%) [20]. Among persons aged over 65, multimorbidity and frailty increase the risk of the financial burden of healthcare as well as the presence of some chronic disease or cancer does, when in need of more contact with the health system and prescribed medicines [4,6,7,8,21,22,23,24]. There appears to be no research that has evaluated the older-aged population’s experiences related to the financial burden of medical care, dental care, and medicines in Slovenia, Serbia, and Croatia using specific and comparable data. The majority of previous studies have examined the economic effects, most often related to some specific diseases or conditions.

This study aimed to determine the frequency of older-aged persons who had the financial burden due to using medical care, dental care, and medicines. The second aim was to determine the characteristics of households and household members (65+ years old) associated with the financial burden of medical care, dental care, and medicines. We hypothesized that the older-aged people in Slovenia, Serbia, and Croatia are well protected from the financial burden of healthcare expenses because these three countries have almost universal health insurance coverage and because retired persons, most often 65+ years old, are excluded from paying contributions while fully covered with health insurance.

## 2. Materials and Methods

### 2.1. The Setting, Data Source, and Study Design

This research represents a secondary analysis of the data from Eurostat, the 2017 European Union Survey on Statistics on Income and Living Conditions (EU-SILC). EU-SILC provides comparable cross-sectional and longitudinal data on income, poverty, social exclusion, housing, labor, education, and health [3]. The survey was launched in 2003 in six EU member states for the first time. The 2017 wave was conducted in 35 European countries, including 28 EU countries in that year, as well as Iceland, Norway, Switzerland, Turkey, Montenegro, North Macedonia, and Serbia. In the 2017 EU-SILC survey, a module on health was conducted. For this purpose, we used data for three countries of former Yugoslavia, available from Eurostat: the Republic of Serbia, the Republic of Slovenia, and the Republic of Croatia [25]. More about EU-SILC data collection, comparability of data, and other questions about the methodology and data quality are available at the Eurostat website [3].

### 2.2. Ethical Consideration

The Ethics Committee from the Faculty of Medicine, University of Belgrade approved the research (No. 1322/VII-9 from 8 July 2021). The permission from European Commission was also obtained (Ref. Ares(2019)6720595 from 30 October 2019).

### 2.3. Sample Design and Participants Selection

Based on Eurostat methodology, the observation units, in all three countries, were households with all household members. A two-stage, stratified rotating panel was used to form the sample. The 2011 Census circles were used as a primary unit in Serbia and Croatia, and in Slovenia, it was the 2016 Central Population Register. Primary units were stratified by population density (Serbia and Croatia) or agricultural density (Slovenia) and, according to the territory, in regions. The second-stage units, in all three countries, were households and all household members. Persons living in collectives (monasteries, nursing homes, military institutions, prisons, dormitories, etc.) were not covered by the survey [3,26].

In Serbia, the sample consisted of 5263 households with 16,659 household members. In Slovenia, the sample consisted of 8801 households with 26,306 household members, and in Croatia, 7842 households with 20,099 household members. The response rate at the household level in Serbia was 86.1%, in Slovenia, 73.9%, and in Croatia, 74.1%. The original dataset for these three countries was made of a total of 21,906 households with 63,064 household members. All individuals who were 65 and more years old on the date when interviewed were considered in this study. This age group was chosen for the study because older-aged people in developed economies are commonly defined as those aged 65 years or more [21]. The final study sample consisted of 12,900 individuals: 4565 from Slovenia, 3424 from Serbia, and 4911 from Croatia.

For this study, inclusion criteria were (i) households with members who were 65 years or more from Slovenia, Serbia, and Croatia and (ii) household members aged 65 years or more. The exclusion criteria were (i) persons living in collectives (monasteries, nursing homes, military institutions, prisons, dormitories, etc.) and (ii) persons who were aged below 65 years.

### 2.4. Questionnaires

In the EU-SILC survey, two questionnaires were used, a household questionnaire and a personal questionnaire. The household questionnaire contained questions referring to the housing, household type, incomes, costs, and financial burdens (related to healthcare and housing costs). The individual questionnaire contained questions related to gender, age, education, marital status, employment and labor market status, self-rated general health, chronic illness, limitations in activities due to health problems, body mass index (BMI), lifestyle, utilization of healthcare, and unmet healthcare needs [3].

### 2.5. Variables Selection

#### 2.5.1. Dependent Variables

In this research, three dependent variables related to the financial burden of healthcare were identified: the financial burden of medical care, the financial burden of dental care, and the financial burden of medicines. Questions about the financial burden of healthcare were part of the household questionnaire. Answers were applied at the household level referring to the household as a whole [3]. In that way, the financial burden of healthcare, if present, was affecting all the members of the household.

Medical care refers to examinations or treatments provided by or under the direct supervision of medical doctors or other medical professionals, including curative, rehabilitative, long-term healthcare, inpatient, outpatient, day and home care, medical mental healthcare, and preventive medical services [3]. Dental care refers to examinations or treatments provided by or under the direct supervision of dentists and orthodontists and preventive dental services [3]. Medicines were defined as products that were used to alleviate symptoms, to prevent illness, or to improve poor health, including medicines prescribed by a doctor or dentist (irrespective of whether they are reimbursed by health insurance or not), nonprescribed medicines (over-the-counter medicines), medicines used at the respondent’s initiative or following consultation with a doctor but not written on a prescription, herbal medicines (excluding herbal teas not considered as medicines), homeopathic medicines, dietary supplements (vitamins, minerals, or tonics), contraceptive pills used for purposes other than contraception (contraception purpose was excluded), and other hormones [3]. For this research, these three variables were computed into binary variables as shown in Table 1.

#### 2.5.2. Independent Variables

Independent variables were classified into two groups: individual-level variables and household-level variables. The nine individual-level variables in this research were the following: sex (male/female); age (computed as age groups: 65–69 years/70–74 years/75–79 years/80 and more than 80 years); marital status (re-coded into “single” for those never married, separated, divorced or widowed, and “married or cohabiting”); self-perceived general health status (very good/good/fair/bad/very bad); the presence of any chronic, long-standing illness or condition (no/yes); limitations in daily activities due to health problems (not limited at all/limited but not severely/severely limited); education level (re-coded into primary school or less/secondary education including lower, upper, postsecondary nontertiary/tertiary education); the number of years spent in paid work (in the number of years), and at the risk of poverty or social exclusion rate (no/yes).

At the risk of poverty or social exclusion rate (AROPE) is the share of the total population at risk of poverty or social exclusion. It is the sum of persons who are either at risk of poverty or are severely materially and socially deprived or living in a household with a very low work intensity [27]. This variable was recoded into “no” (for those participants without risk of poverty, without severe material deprivation, and without low work intensity) and “yes” (if at least one risk—risk of poverty, severe material deprivation, or low work intensity—was reported).

The four variables at the household level were as follows: household’s size (recoded as one person/two persons/three and more persons); financial burden of total housing costs (not a burden at all/a slight burden/a heavy burden); ability to make ends meet (re-coded into easily or very easily/with some difficulty or fairly easily/with difficulty or with great difficulty); severe material deprivation rate (not severely deprived/severely deprived).

Severe material deprivation rate shows “an enforced lack of necessary and desirable items to lead an adequate life”, calculated as the proportion of the population that cannot afford at least 4 out of 9 predefined material items considered by most people to be desirable or even necessary to lead an adequate life (presence of arrears on mortgages or rent payments, on utility bills, on hire purchase installments or other loans; capacity to afford to pay for one week holiday away from home; capacity to afford a meal with meat, chicken, fish (or vegetarian equivalent) every second day; capacity to face unexpected financial expenses; possession of telephone, color TV, washing machine, car, and possession of a heating system to keep home adequately warm [28]. The ability to make ends meet assesses the respondents’ feelings about the level of difficulty experienced by the household in making ends meet [28]. All “net” income sources, by all household members, were taken into account. The objective of assessing the financial burden of the total housing costs was the respondent’s feeling about the extent to which housing costs were a financial burden to the household. Only those housing costs that were paid had been taken into account [3].

The study complies with the protocol, instruments, and methodological guidance of Eurostat. Detailed official information about the EU-SILC survey and answers to questions concerning the quality and comparability of data are freely provided by Eurostat [3].

Results of the Survey were published on an aggregate level, and the anonymity of interviewed individuals and households is fully secured. The responsibility for all conclusions drawn from the data lies entirely with the authors.

### 2.6. Statistical Analysis

To analyze participants’ and households’ characteristics, descriptive statistics were performed, using the absolute numbers and frequencies (for the qualitative variables) or mean and standard deviation (for the quantitative variables). The significance of the associations between potential explanatory variables (on the personal and household level) and the financial burden of medical care, dental care, and medicines (as the study outcome variable) was assessed through the Pearson’s chi-squared test and Mann–Whitney test. All variables, personal and household, found to be significantly associated with the financial burden of medical care, dental care, and medicines were included in the multivariate logistic regression models, for each country separately.

Two multivariate logistic regression models were performed for each of the three financial burdens of healthcare (medical, dental, and medicines). In Model 1, only variables expressing the personal characteristics of the participants were used, while in Model 2, both variables expressing personal and household characteristics were used. These models identified factors in the three observed countries that explained the presence of the financial burden of medical care, dental care, and medicines, with their odds ratio (OR) and with the 95% confidence interval (95% CI). For categorical variables, the OR was presented regarding the reference category, and for continuous variables, the OR represented the increase in odds of the financial burden of medical care, dental care, and medicines with every one-unit increase of the input variable.

The results were considered statistically significant when the *p*-value was less than 0.05 for all performed analyses. The statistics were performed using the Statistical Package for the Social Sciences (SPSS) software version 22 (SPSS 22.0 for Windows, SPSS Inc., IBM, Armonk, NY, USA).

## 3. Results

### 3.1. Individual and Household Characteristics of the Participants

There were 12,900 participants, 35.4% (4565) were from Slovenia, 26.5% (3424) from Serbia, and 38.1% (4911) from Croatia. Respondents were aged on average 73.2 years (SD = 5.4), most of them in the age group of 65–69 years (33.1%; 4272), and most of them were women (56.3%; 7264), married or cohabiting (58.2%; 7499). Characteristics of the participants and households, in all three countries and in total, are presented in Table 2.

### 3.2. Frequency of Financial Burden of Healthcare and Characteristics of the Participants

As shown in Table 3, Table 4 and Table 5, participants in Croatia had the highest share of the financial burden of medical care and of medicines (2167; 50.0% and 3003; 69.1%, respectively) and participants in Slovenia had the highest share for the financial burden of dental care (1457; 48.5%). These differences in the number of participants with and without the financial burden of medical care, dental care, and medicines in the observed countries were statistically significant (*p* < 0.01).

Results for Slovenia showed there was a significant (*p* < 0.05) difference between the participants with and without the financial burden of medical care in age, marital status, limitation in activity because of health problems, level of education, number of years spent in paid work, and risk of poverty and social exclusion, and on the household level, in household size and financial burden of total housing costs. Participants from Serbia, with and without the financial burden of medical care, statistically significantly differed (*p* < 0.05) in age, marital status, general health, presence of chronic illness or conditions, limitation in activity because of health problems, level of education, and risk of poverty and social exclusion, and on the household level, in household size, ability to make ends meet, material deprivation of the households, and financial burden of total housing costs. In Croatia, there was a significant (*p* < 0.05) difference between the participants with and without the financial burden of medical care in marital status, general health, presence of chronicle illness or conditions and limitation in activity because of health problems, and on the household level, in the financial burden of total housing costs and ability to make ends meet (Table 3).

In Slovenia, there was a significant (*p* < 0.05) difference between the participants with and without the financial burden of dental care in sex, age, marital status, level of education, and number of years spent in paid work, and on a household level, in household size, the financial burden of total housing costs, and ability to make ends meet. Participants from Serbia, with and without the financial burden of dental care, significantly (*p* < 0.05) differed in general health, limitation in daily activity because of health problems, and risk of poverty or social exclusion, and on the household level, in the ability to make ends meet, in material deprivation of the households, and the financial burden of total housing costs. There was a significant (*p* < 0.05) difference between the participants with and without the financial burden of dental care in general health, limitation in daily activity because of health problems, marital status, and education level, and on a household level, in household size, the financial burden of total housing costs, and in the ability to make ends meet (Table 4).

There was a significant (*p* < 0.05) difference between the participants with and without the financial burden of medicines in all examined characteristics except sex, age, and marital status in Slovenia, except sex, age, and the number of years spent in paid work in Serbia, and except marital status and the number of years spent in paid work in Croatia (Table 5).

### 3.3. The Effects of Individual and Household Characteristics on Financial Burden of Medical Care, Dental Care and Medicines in Slovenia, Serbia, and Croatia

Marital status (in Slovenia and Croatia), self-reported general health as bad and very bad (Serbia and Croatia), limitation in activity because of health problems, and education level (both for Slovenia) were associated with the financial burden of medical care, as were the heavy financial burden of total housing costs (all three countries) and ability to make ends meet with any level of difficulty (Serbia and Croatia). These results are shown in Table 6.

Multivariate logistic regression models for the financial burden of dental care, presented in Table 7, show that marital status (in Slovenia and Croatia, on a personal level), high (tertiary) education level (in Slovenia and Croatia), number of years spent in paid work (for Slovenia), presence of risk of poverty or social exclusion (Serbia, personal level), the financial burden of total housing costs (all three countries) and ability to make ends meet (Slovenia and Croatia) were associated with the financial burden of dental care (*p* < 0.05).

### 3.4. The Effects of Individual and Household Characteristics on Financial Burden of Medicines in Slovenia, Serbia, and Croatia

Multivariate logistic regression models for the financial burden of medicines, presented in Table 8, show that age from 70 to 74 years old (in Serbia), marital status (Croatia), self-reported general health (for all three countries but with differences shown in Table 8), presence of chronic illness or condition (Serbia and Croatia, on a personal level), presence of limitation in daily activity because of health problems (Slovenia and Croatia), presence of risk of poverty or social exclusion (Serbia, on personal level, and Croatia), household size (Slovenia), the financial burden of total housing costs and ability to make ends meet (all three countries), and severe material deprivation of household (Serbia) were associated with the financial burden of medicines.

## 4. Discussion

This study provides evidence that the financial burden of healthcare (medical, dental care, or medicines) is present in Slovenia, Serbia, and Croatia, but in different frequencies. One-third of participants 65 or more years old reported financial burden of medical care. More than that, about two-fifths reported the financial burden of dental care and more than two-thirds reported the financial burden of medicines.

Among the EU-28 countries in 2017, 44.3% of participants in the EU-SILC study reported some level of the financial burden of medical care, 50.7% reported financial burden of dental care, and 50.4% that of medicines [29]. Compared with that data, older-aged participants in this study reported a higher percentage of the financial burden for medical care only in Croatia. In all of the observed countries, the financial burden of dental care was less frequent, while the financial burden for medicines was more frequent than average in EU-28 countries in 2017 [29]. These differences could be associated with health insurance policies, socio-economic characteristics, healthcare needs, quality of healthcare, and economic development of the country [1,2,4,5,12,19,30]. It was noticed that medicines were the main drivers of the financial burden of healthcare in the WHO European region, followed by spending on inpatient care and dental care [31], and that financial hardship due to the out-of-pocket payments on medicines are more likely in health systems where financial protection is weaker, and on dental care where financial protection is stronger [4].

According to the study results, the financial burden of medical care was more frequent in Croatia than in the other two countries, as was the financial burden of medicines, while the financial burden of dental care was more frequent in Slovenia. In the observed countries, population health insurance coverage is almost universal and citizens are covered with a wide range of medical services [9,10,11]. Differences among these countries’ insurance policies refer to the type and extent of benefits and reductions in patients’ charges for medical care. People aged 75 and more in Slovenia are exempt from co-payments for medical care, while in Serbia and Croatia, there are no exemptions for older-aged people unless associated with low income or chronic diseases or disability [9,10,11,32,33]. According to the previous research, the financial burden of dental care is caused by gaps in the insurance coverage [34], which are widespread in Europe, resulting in unmet needs for poorer people (avoiding dental care due to costs) and financial hardship for richer people (due to paying dental care costs) [4]. Prosthetic treatment is more frequently required among the older-aged [35], and it is partially covered by public insurance for people over 65 years but with different beneficiary policies [9,10,11]. These differences could be one of the reasons for the disparity in the percentage of old-aged people facing the financial burden of dental care in these countries. Another reason could be the use of private practice for dental services [9,10,11].

In our study, the financial burden of medicines was more frequent in Croatia than in the other two countries, but in these three countries, the financial burden of medicines was more frequent among older-aged people compared to the average for the country (Slovenia 47.9%, Serbia 41.2%, and Croatia 44.7%) [29]. Public insurance policy and reduction in co-payments for medicines are protective measures regarding the financial burden of medicines [4,36]. In Slovenia, Serbia, and Croatia, compulsory health insurance covers the costs for medicines on positive lists (specific for the country) but some co-payments are required [9,10,11,32,33,37]. Most European countries apply reduction or exemption mechanisms for outpatient medicines included in the benefits package scheme [36]. The results in this study for Serbia show that participants from 70 to 74 years of age have higher odds of the financial burden of medicines than other age groups, and previous analysis of out-of-pocket payments in Serbia showed that the implementation of the exemption mechanism in Serbia has failed, in particular for the older-aged people (over 65 years) and the poor (low family income and unemployed) [38].

The financial burden of healthcare among older-aged people in these countries may be associated with their socio-economic and health characteristics (1,4,5,6,19). This study found that being married or cohabiting is associated with the likelihood of having a financial burden of medical care (Slovenia and Croatia), dental care (Slovenia), and medicines (Croatia). A possible explanation for these findings could be that doubling the size of expenses for healthcare could lead households, with two older-aged spouses, to a financial burden. Moreover, it might be that one spouse was unemployed through the life course, is without pension income, and is financially dependent on the other one [21]. It is noticed that people living in multigenerational households face the highest rates of the financial burden of healthcare [30,39]. This explanation correlates with other results from this study. Namely, older-aged participants living in households of three or more people in Slovenia were found to have higher odds of the financial burden of medicines.

Poorly assessed general health was associated with the likelihood of reporting the financial burden of medical care (in Serbia), and medicines (in Serbia and Croatia) in this study. In addition, participants from Serbia who suffered from any chronic illness or condition had higher odds of having the financial burden of medicines, the same as participants from Slovenia and Croatia who reported limitations in daily activities due to health problems. In addition, participants from Slovenia with limitations in daily activities due to health problems were more likely to have the financial burden of medical care. Previous research indicated that the number of chronic diseases increases with age [40], as does the limitation in daily activities due to health problems [41]. An increasing number of chronic diseases was associated with worse self-reported general health [40] and was in positive correlation with the number of visits in primary and secondary settings among the older-aged [6,21,40,41]. The likelihood of the financial burden of healthcare is present when both the level of healthcare use and out-of-pocket payments as a percentage of health expenditure are high [30]. Out-of-pocket expenditure as a percentage of health expenditure in 2017, in the general population, was the highest in Serbia (40.6%), compared with Slovenia and Croatia (11.0% and 12.3%, respectively) [13]. All of the previously mentioned factors could be the reasons for higher odds of the financial burden of medical care and medicines. If health insurance covers a wide range of inpatient and outpatient services, but not medicines prescribed during the visits, insurance coverage may lead to a higher financial burden because households need to pay for medicines [30]. Even in high-income countries, including Slovenia, those with health insurance but with health problems were more exposed to out-of-pocket costs compared to those with good health [19], as was the case with the poorest and in households with older-aged people [42]. Some unexpected circumstances, such as the COVID-19 pandemic, significantly worsen financial protection globally, affecting the older-aged population and poor households [43].

According to the study results for Slovenia, the likelihood of facing the financial burden of medical and dental care was higher among those with high (tertiary) education compared with those with secondary education. Additionally, for Slovenia, the likelihood of having the financial burden of dental care was higher for each year spent in paid work. Previous results show that older people with lower educational level and lower income are most likely not to pay any out-of-pocket costs for dental care, and if out-of-pocket payments are made, the amounts are greater for those with higher income and with a higher level of education [44].

Several indicators of poverty, social exclusion, and material deprivation are included in this study. According to the study results, these indicators are in association with the likelihood of the financial burden of healthcare. A previous study found that older people represent the vulnerable group in society, with an increased risk of poverty or social exclusion [21,30,43]. The results from this study show that older-aged people with risk of poverty or social exclusion had a lower likelihood of financial burden of medicines in Croatia. In addition, participants from each of these three countries who reported the financial burden of total housing costs had lower odds for the financial burden of medical care, dental care, and medicines. One of the reasons for these findings could be the changes in the structure of household consumption expenditure. Households with older people usually have a lower-than-average level of consumption expenditure, considering new housing and personal items luxury in comparison with healthcare [21]. Another reason could be found in health insurance policies in these countries, health insurance coverage, and beneficiary policy for older-aged and low-income population groups [9,10,11]. Some other research results indicate opposite explanations. Namely, it was found that the financial burden of healthcare could increase avoidance of seeking healthcare, and in that case, people do not report the financial burden of healthcare [4].

This study has several strengths and limitations. To the best of our knowledge, this is the first study that compares the financial burden of medical care, dental care, and medicines in Slovenia, Serbia, and Croatia. Comparison of healthcare systems between countries could lead to the transposition of good practices identified in the other counties [45]. Using nonmonetary measures of the financial burden is an additional value of this study. Moreover, our findings are based on a large sample of older-aged individuals from a nationally representative sample [3].

One of the limitations is the cross-sectional design of the study with constrained possibility for precisely underlining causality of risk factors and financial burden of health, especially because questions about the financial burden were related to the healthcare expenses in the previous year, which may have led to recall bias and underreporting. Another limitation is the sampling design that does not include older-aged people living in collectives (monasteries, nursing homes, military institutions, prisons, dormitories, etc.). Information about the financial burden of healthcare was collected at the household level and reflected the financial burden of all family members [3], and it was not possible to conclude which family members generate the most medical costs, possibly higher than that of the examined older-aged members. However, from the family perspective, having one family member who contributes to the financial burden for healthcare may place the entire family into financial strains [39]. There are no questions about health insurance included in the EU-SILC 2017 survey [3], therefore it lacks information about protective mechanisms for the financial burden of healthcare in Slovenia, Serbia, and Croatia. Namely, besides the compulsory insurance in these countries voluntary health insurance is available [9,10,11], and it was reported that families with a mixture of coverage types within the family members, and families with uninsured members were more likely to experience the financial burden of medical care than were families in which all members had either private or public coverage [39]. Furthermore, questions about the financial burden of healthcare referred only to the out-of-pocket expenditure at the point of use, while costs of compulsory or voluntary health insurance were excluded [3]. The use of private practice for medical and dental care was not the subject of this study, representing another study limitation. There is no comparable data on expenses that older-aged people have regarding visits to private practice in observed countries. These expenses are more often out of the range of compulsory health insurance, and patients have to pay the full price by themselves, including that of the medicines prescribed during the visits, except if the private provider is contracted with compulsory health insurance, such as the case for some primary healthcare doctors in Slovenia [9] and privatized primary healthcare in Croatia [11] or some services in Serbia (e.g., dialysis and cataract surgery) [46]. The increasing need for long-term services and the expenses for that kind of care were not included in the study, but could influence healthcare and overall households’ costs.

Generalizing questions on the financial burden of healthcare without causality separation (health insurance, out-of-pocket payments, formal or informal, expenses for private practice visits, hospital care, etc.) do not give deep insight into the structure of the financial burden. Further studies focusing on these observations could give better scope on the structure of financial burden of healthcare and its causality with socio-economic characteristics of the population, type, and health insurance coverage extension and health systems characteristics.

## 5. Conclusions

It is evident that compulsory health insurance provides financial protection against high healthcare costs, but it seems that further tailoring is needed. Namely, some population groups could be neglected or some health needs could be unmet. The growing older population and their healthcare needs might be invisible to decision makers and left under the risk of the financial burden of healthcare. This study shows that the financial burden of healthcare is present among the older-aged population in Slovenia, Serbia, and Croatia and that socio-economic and health characteristics are associated with the likelihood of financial burden. The financial burden of medical care and medicines was the most frequent among older-aged people in Croatia, and for dental care, in Slovenia. The highest likelihood of financial burden of medical care was found in Serbia, among participants who make their ends meet with different levels of difficulty, and among those with very bad and bad self-reported general health. The highest likelihood for the financial burden of dental care was in Croatia, among those who make their ends meet with difficulty or with great difficulty, and in Slovenia, among those who make their ends meet fairly easily or with some difficulty. The financial burden of medicines had the highest odds among older-aged people who make their ends meet with difficulty or with great difficulty in Serbia and in Croatia. Additionally, the odds of financial burden of medicines were higher among older-aged people with very bad self-reported general health in Serbia compared to those with very good health. At the highest risk of the financial burden of healthcare were the poorest older-aged people living in households with the financial difficulties for everyday expenses and those with bad self-reported general health.

This study does not explore the causes of financial burden and whether, or not, it was because of the out-of-pocket payments for services and goods not included in the health insurance. This research brings insight into existing problem for the older-aged population which is under the high risk of the financial burden of healthcare but should be protected against it. Further works should give the in-depth causality of the financial burden of healthcare, and that could be used by policymakers to adopt health insurance protective measures for the most vulnerable people.

## Figures and Tables

**Table 1:** The financial burden of healthcare—dependent variables.

Variable Name	Questioned as:	Original Answers	Computed Variable
Financial burden of medical care	“To what extent were the costs of medical examinations or treatments a financial burden to your household during the past 12 months?”	1—a heavy burden 2—somewhat a burden 3—not a burden at all	0—without financial burden 1—with financial burden (including the original answers “heavy burden” and “somewhat burden”).
Financial burden of dental care	“To what extent were the costs of dental examinations or treatments a financial burden to your household during the past 12 months?”	1—a heavy burden 2—somewhat a burden 3—not a burden at all	0—without financial burden 1—with financial burden (including the original answers “heavy burden” and “somewhat burden”).
Financial burden of medicine	“To what extent were the costs of medicines (prescribed and nonprescribed) a financial burden to your household during the past 12 months?”	1—a heavy burden 2—somewhat a burden 3—not a burden at all	0—without financial burden 1—with financial burden (including the original answers “heavy burden” and “somewhat burden”).

Note: variable names, questions, and original answers were taken from Eurostat—EU statistics on income and living conditions (EU-SILC) methodology, available at https://ec.europa.eu/eurostat/web/income-and-living-conditions/methodology (accessed on 8 March 2022).

**Table 2:** Individual and household characteristics of the older-aged participants from Slovenia, Serbia, and Croatia.

Participants’ Characteristics	Slovenia		Serbia		Croatia		Total	
	n	%	*n*	%	n	%	*n*	%
	4565	35.4	3424	26.5	4911	38.1	12,900	100.0
Sex								
male	2078	45.5	1476	43.1	2082	42.4	5636	43.7
female	2487	54.5	1948	56.9	2829	57.6	7264	56.3
Total	4565	100.0	3424	100.0	4911	100.0	12,900	100.0
Age group								
65–69	1600	35.0	1152	33.6	1520	31.0	4272	33.1
70–74	1073	23.5	795	23.2	1178	24.0	3046	23.6
75–79	885	19.4	731	21.3	1047	21.3	2663	20.6
80 and more	1007	22.1	746	21.8	1166	23.7	2919	22.6
Total	4565	100.0	3424	100.0	4911	100.0	12,900	100.0
Marital status								
single/separated/divorced/widowed	1586	34.9	1784	52.1	2156	43.9	5382	41.8
married/cohabit	2960	65.1	1640	47.9	2755	56.1	7499	58.2
Total	4546	100.0	3424	100.0	4911	100.0	12,881	100.0
Self-perceived general health status								
very good	80	3.6	54	1.6	105	2.1	239	2.3
good	637	28.4	437	12.8	876	17.9	1950	18.4
fair	966	43.1	1328	38.8	1682	34.3	3976	37.6
bad	464	20.7	1322	38.6	1718	35.0	3504	33.1
very bad	96	4.3	283	8.3	523	10.7	902	8.5
Total	2243	100.0	3424	100.0	4904	100.0	10,571	100.0
Suffer from any chronic illness or condition								
no	1534	68.4	2416	70.6	3647	74.4	7597	71.9
yes	709	31.6	1008	29.4	1256	25.6	2973	28.1
Total	2243	100.0	3424	100.0	4903	100.0	10,570	100.0
Limitations in daily activities due to health problems								
not limited at all	494	22.0	467	13.6	1256	25.6	2217	21.0
limited but not severely	922	41.1	991	28.9	2155	43.9	4068	38.5
severely limited	827	36.9	1966	57.4	1493	30.4	4286	40.5
Total	2243	100.0	3424	100.0	4904	100.0	10,571	100.0
Education								
primary school or less	0	0.0	1121	32.7	802	16.3	1923	14.9
secondary	3899	85.4	1816	53.0	3480	70.9	9195	71.3
high/tertiary	666	14.6	487	14.2	629	12.8	1782	13.8
Total	4565	100.0	3424	100.0	4911	100.0	12,900	100.0
Number of years spent in paid work arithmetic mean ± standard deviation	34.5 ± 2.0		33.6 ± 2.6		32.3 ± 4.1		33.2 ± 8.7	
At the risk of poverty or social exclusion								
no	3903	85.5	2306	67.3	3243	66.0	9452	73.3
yes	662	14.5	1118	32.7	1668	34.0	3448	26.7
Total	4565	100.0	3424	100.0	4911	100.0	12,900	100.0
Household size								
one person	686	15.0	686	20.0	1337	27.2	2709	21.0
two persons	2292	50.2	1113	32.5	2452	49.9	5857	45.4
three or more persons	1587	34.8	1625	47.5	1122	22.8	4334	33.6
Total	4656	100.0	3424	100.0	4911	100.0	12,900	100.0
Financial burden of total housing costs								
not a burden at all	1580	34.6	2184	63.9	2643	54.7	6407	50.0
a slight burden	2391	52.4	1112	32.5	1882	38.9	5385	42.0
a heavy burden	594	13.0	124	3.6	310	6.4	1028	8.0
Total	4565	100.0	3420	100.0	4835	100.0	12,820	100.0
Ability to make ends meet								
easily or very easily	673	14.7	59	1.7	166	3.4	898	7.0
fairly easily or with some difficulty	2799	61.3	1233	36.0	2376	48.4	6408	49.7
with difficulty or with great difficulty	1093	23.9	2132	62.3	2367	48.2	5592	43.4
Total	4565	100.0	3424	100.0	4909	100.0	12,898	100.0
Severely materially deprived household								
not severely deprived	4356	95.4	2770	80.9	4245	86.4	11,371	88.1
severely deprived	209	4.6	654	19.1	666	13.6	1529	11.9
Total	4565	100.0	3424	100.0	4911	100.0	12,900	100.0

*n*—number of respondents.

**Table 3:** Distribution of participants with and without the financial burden of medical care in Slovenia, Serbia, and Croatia.

	Slovenia					Serbia			Croatia		
Participants’ Characteristics	No	Yes				No	Yes		No	Yes	
	*n* = 3519 (84.9%)	*n* = 626 (15.1%)		*p*		*n* = 1739 (60.3%)	*n* = 1143 (39.7%)	*p*	*n* = 2166 (50.0%)	*n* = 2167 (50.0%)	*p*
PERSONAL LEVEL											
Sex											
male	1594 (45.3)		301 (48.1)		0.213	776 (44.6)	475 (41.6)	0.113	908 (41.9)	934 (43.1)	0.450
female	1925 (54.7)		325 (51.9)			963 (55.4)	668 (58.4)		1258 (58.1)	1233 (57.5)	
Age group											
65–69	1197 (34.0)		240 (38.3)		0.038	621 (35.7)	341 (29.8)	0.011	695 (32.1)	634 (29.3)	0.180
70–74	819 (23.3)		153 (24.4)			390 (22.4)	286 (25.0)		525 (24.2)	525 (24.2)	
75–79	699 (19.9)		118 (18.8)			377 (21.7)	260 (22.7)		443 (20.5)	480 (22.2)	
80+	804 (22.8)		115 (18.4)			351 (20.2)	256 (22.4)		503 (23.2)	528(24.4)	
Marital status											
single/separated/ divorced/widowed	1243 (35.3)		155 (24.8)		<0.01	796 (45.8)	528 (46.2)	0.854	994 (45.9)	853 (39.4)	<0.01
married/cohabit	2276 (64.7)		741 (75.2)			943 (54.2)	615 (53.8)		1172 (54.1)	1314 (60.6)	
Self-perceived general health status											
very good	51 (2.9)		10 (3.3)		0.411	33 (1.9)	6 (0.5)	<0.01	41 (1.9)	29 (1.3)	<0.01
good	488 (27.9)		70 (23.4)			255 (14.7)	94 (8.2)		443 (20.5)	287 (13.3)	
fair	758 (43.4)		133 (44.5)			762 (43.8)	334 (29.2)		755 (34.9)	704 (32.5)	
bad	368 (21.2)		74 (24.7)			595 (34.2)	556 (48.6)		707 (32.7)	867 (40.1)	
very bad	81 (4.6)		12 (4.0)			94 (5.4)	153 (13.4)		216 (10.0)	277 (12.8)	
Suffer from any chronic illness or condition											
no	519 (29.7)		79 (26.4)		0.275	521 (30.0)	246 (21.5)	<0.01	532 (24.6)	460 (21.3)	<0.01
yes	1227 (70.3)		220 (43.6)			1218 (70.0)	897 (78.5)		1630 (75.4)	1704 (78.7)	
Limitations in daily activities due to health problems											
not limited at all	626 (35.9)		85 (28.4)		0.030	1063 (61.1)	551 (48.2)	<0.01	712 (32.9)	562 (26.0)	<0.01
limited but not severely	728 (41.7)		133 (44.5)			491 (28.2)	370 (32.4)		912 (42.2)	981 (45.3)	
severely limited	392 (22.5)		81 (27.1)			185 (10.6)	222 (19.4)		538 (24.9)	621 (28.7)	
Education											
primary school or less	0		0			517 (29.7)	424 (37.1)	<0.01	334 (15.4)	352 (16.2)	0.427
secondary	3039 (86.4)		498 (79.6)		<0.01	949 (54.6)	583 (51.0)		1538 (71.0)	1547 (71.4)	
high/tertiary	480 (13.6)		128 (20.4)			273 (15.7)	136 (11.9)		294 (13.6)	268 (12.4)	
Number of years spent in paid work arithmetic mean ± standard deviation	34.4 ± 8.9		35.3 ± 8.4		<0.01	33.8 ± 11.0	33.5 ± 11.5	0.431	32.4 ± 10.2	32.4 ± 9.9	0.499
At risk of poverty or social exclusion											
no	2997 (85.2)		567 (90.6)		<0.01	1282 (73.7)	685 (59.9)	<0.01	1483 (68.5)	1422 (65.6)	0.050
yes	522 (14.8)		59 (9.4)			457 (26.3)	458 (40.1)		683 (31.5)	745 (34.4)	
HOUSEHOLD LEVEL											
Household size											
one person	540 (15.3)		59 (9.4)		<0.01	315 (18.1)	213 (18.6)	0.389	595 (27.5)	528 (24.4)	0.064
two persons	1741 (49.5)		377 (60.2)			595 (34.2)	363 (31.8)		1091 (50.4)	1131 (52.2)	
three or more persons	1238 (35.2)		190 (30.4)			829 (47.7)	567 (49.6)		480 (22.2)	508 (23.4)	
Financial burden of total housing costs											
not a burden at all	479 (13.6)		52 (8.3)		<0.01	965 (55.5)	868 (76.1)	<0.01	185 (8.7)	83 (3.9)	<0.01
a slight burden	1814 (51.5)		362 (57.8)			686 (39.5)	258 (22.6)		806 (37.7)	826 (38.7)	
a heavy burden	1226 (34.8)		212 (33.9)			87 (5.0)	15 (1.3)		1147 (53.6)	1226 (57.4)	
Ability to make ends meet											
easily or very easily	508 (14.4)		81 (12.9)		0.598	46 (2.6)	3 (0.3)	<0.01	106 (4,9)	41 (1,9)	<0.01
fairly easily or with some difficulty	2168 (61.6)		395 (63.1)			785 (45.1)	260 (22.7)		1108 (51,2)	1005 (46,4)	
with difficulty or with great difficulty	843 (24.0)		150 (24.0)			908 (52.2)	880 (77.0)		952 (44,0)	1121 (51,7)	
Severely materially deprived household											
not severely deprived	3363 (95.6)		603 (96.3)		0.451	1511 (86.9)	854 (74.7)	<0.01	1896 (87.5)	1840 (84.9)	<0.01
severely deprived	156 (4.4)		23 (3.7)			228 (13.1)	289 (25.3)		270 (12.5)	327 (15.1)	

*n*—number of the respondents; *p*—*p*-value.

**Table 4:** Distribution of participants with and without the financial burden of dental care in Slovenia, Serbia, and Croatia.

Participants’ Characteristics	Slovenia			Serbia			Croatia		
	No	Yes		No	Yes		No	Yes	
	*n* = 1546 (51.5%)	*n* = 1457 (48.5%)	*p*	*n* = 714 (53.3%)	*n* = 626 (46.7%)	*p*	*n* = 2.004 (63.6%)	*n* = 1146 (36.4%)	*p*
PERSONAL LEVEL									
Sex									
male	704 (45.5)	717 (49.2)	0.048	333 (46.6)	263 (42.0)	0.100	867 (43.3)	508 (44.3)	0.588
female	842 (54.5)	740 (50.8)		381 (53.4)	363 (58.0)		1137 (56.7)	638 (55.7)	
Age group									
65–69	539 (34.9)	599 (41.1)	<0.01	270 (37.8)	222 (35.5)	0.245	665 (33.2)	381 (33.2)	0.713
70–74	386 (25.0)	326 (22.4)		175 (24.5)	138 (22.0)		497 (24.8)	284 (24.8)	
75–79	283 (18.3)	286 (19.6)		139 (19.5)	148 (23.6)		406 (20.3)	248 (21.6)	
80+	338 (21.9)	246 (16.9)		130 (18.2)	118 (18.8)		436 (21.8)	233 (20.3)	
Marital status									
single/separated/ divorced/widowed	539 (34.9)	408 (28.0)	<0.01	297 (41.6)	283 (45.2)	0.202	851 (42.5)	418 (36.5)	<0.01
married/cohabiting	1007 (65.1)	1049 (72.0)		417 (58.4)	343 (54.8)		1153 (57.5)	728 (63.5)	
Self-perceived general health status									
very good	29 (4.2)	21 (3.3)	0.340	13 (1.8)	11 (1.8)	<0.01	40 (2.0)	18 (1.6)	0.302
good	199 (28.6)	201 (31.6)		117 (16.4)	60 (9.6)		403 (20.2)	199 (17.4)	
fair	301 (43.2)	274 (43.0)		332 (46.5)	237 (37.9)		694 (34.7)	406 (35.5)	
bad	132 (19.0)	121 (19.0)		212 (29.7)	267 (42.7)		683 (34.2)	412 (36.0)	
very bad	35 (5.0)	20 (3.1)		40 (5.6)	51 (8.1)		178 (8.9)	110 (9.6)	
Suffer from any chronic illness or condition									
no	213 (30.6)	206 (32.3)	0.533	248 (34.7)	154 (24.6)	<0.01	527 (26.4)	273 (23.8)	0.127
yes	483 (69.4)	431 (67.7)		466 (65.3)	472 (75.4)		1471 (73.6)	872 (76.2)	
Limitations in daily activities due to health problems									
not limited at all	274 (39.4)	221 (34.7)	0.159	446 (62.5)	361 (57.7)	0.181	627 (31.4)	339 (29.6)	0.501
limited but not severely	283 (40.7)	289 (45.4)		185 (25.9)	178 (28.4)		889 (44.5)	532 (46.5)	
severely limited	139 (20.0)	127 (19.9)		83 (11.6)	87 (13.9)		482 (24.1)	274 (23.9)	
Education									
primary school or less	0	0		204 (28.6)	200 (31.9)	0.056	283 (14.1)	138 (12.0)	0.037
secondary	1318 (85.3)	1170 (80.3)	<0.01	388 (54.3)	347 (55.4)		1447 (72.2)	818 (71.4)	
high/tertiary	228 (14.7)	287 (19.70		122 (17.1)	79 (12.6)		274 (13.7)	190 (16.6)	
Number of years spent in paid work arithmetic mean ± standard deviation	34.8 ± 8.3	36.0 ± 7.4	<0.01	33.4 ±10.8	34.0 ±10.7	0.446	32.3 ± 10.1	33.0 ± 9.6	0.207
At the risk of poverty or social exclusion									
no	1387 (89.7)	1337 (91.8)	0.062	568 (79.6)	435 (69.5)	<0.01	1424 (71.1)	815 (71.1)	1.000
yes	159 (10.3)	120 (8.2)		146 (20.4)	191 (30.5)		580 (28.9)	331 (28.9)	
HOUSEHOLD LEVEL									
Household size									
one person	171 (11.1)	122 (8.4)	0.038	41 (5.7)	54 (8.6)	0.093	475 (23.7)	220 (19.2)	0.012
two persons	743 (48.1)	737 (50.6)		171 (23.9)	135 (21.6)		1001 (50.0)	596 (52.0)	
three or more persons	632 (40.9)	598 (41.0)		502 (70.3)	437 (69.8)		528 (26.3)	330 (28.8)	
Financial burden of total housing costs									
not a burden at all	215 (13.9)	153 (10.5)	<0.01	342 (47.9)	478 (76.4)	<0.01	181 (9.1)	59 (5.20)	<0.01
a slight burden	891 (57.6)	789 (54.2)		329 (46.1)	141 (22.5)		791 (39.9)	479 (42.2)	
a heavy burden	440 (28.5)	515 (35.3)		43 (6.0)	7 (1.1)		1010 (51.0)	597 (52.6)	
Ability to make ends meet									
easily or very easily	277 (17.9)	182 (12.5)	<0.01	33 (4.6)	0 (0.0)	<0.01	96 (4.8)	31 (2.7)	0.015
fairly easily or with some difficulty	952 (61.6)	997 (68.4)		381 (53.4)	189 (30.2)		1053 (52.5)	606 (52.9)	
with difficulty or with great difficulty	317 (20.5)	278 (19.1)		300 (42.0)	437 (69.8)		855 (42.7)	509 (44.4)	
Severely materially deprived household									
not severely deprived	1496 (96.8)	1423 (97.7)	0.166	663 (92.9)	520 (83.1)	<0.01	1799 (89.8)	1010 (88.1)	0.173
severely deprived	50 (3.2)	34 (2.3)		51 (7.1)	106 (16.9)		205 (10.2)	136 (11.9)	

*n*—number of the respondents; *p*—*p*-value.

**Table 5:** Distribution of participants with and without the financial burden of medicines in Slovenia, Serbia, and Croatia.

Participants’ Characteristics	Slovenia			Serbia			Croatia			
	No	Yes		No	Yes		No	Yes		
	*n* = 1935 (42.4%)	*n* = 2630 (57.6%)	*p*	*n* = 1058 (35.8%)	*n* = 1900 (64.2%)	*p*	*n* = 1342 (30.9%)	*n* = 3003 (69.1%)	*p*	
PERSONAL LEVEL										
Sex										
male	903 (46.7)	1175 (44.7)	0.192	483 (45.7)	790 (41.6)	0.035	562 (41.9)	1279 (42.6)	0.685	
female	1032 (53.3)	1455 (55.3)		575 (54.3)	1110 (58.4)		780 (58.1)	1724 (57.4)		
Age group										
65–69	701 (36.2)	899 (34.2)	0.531	408 (38.6)	582 (30.6)	<0.01	436 (32.5)	887 (29.5)	0.144	
70–74	445 (23.0)	628 (23.9)		219 (20.7)	463 (24.4)		327 (24.4)	731 (24.3)		
75–79	365 (18.9)	520 (19.8)		218 (20.6)	429 (22.6)		264 (19.7)	665 (22.1)		
80+	424 (21.9)	583 (22.2)		213 (20.1)	426 (22.4)		315 (23.5)	720 (24.0)		
Marital status										
single/separated/ divorced/widowed	698 (36.1)	888 (33.8)	0.109	490 (46.3)	879 (46.3)	1.000	651 (48.5)	1199 (39.9)	<0.01	
married/cohabit	1237 (63.9)	1742 (66.2)		568 (53.7)	1021 (53.7)		691 (51.5)	1804 (60.1)		
Self-perceived general health status										
very good	52 (5.3)	28 (2.2)	<0.01	22 (2.1)	14 (0.7)	<0.01	37 (2.8)	33 (1.1)	<0.01	
good	351 (35.9)	286 (22.6)		184 (17.4)	178 (9.4)		332 (24.8)	399 (13.3)		
fair	425 (43.5)	541 (42.8)		520 (49.1)	620 (32.6)		505 (37.7)	966 (32.2)		
bad	132 (13.5)	332 (26.2)		290 (27.4)	877 (46.2)		359 (26.8)	1215 (40.5)		
very bad	18 (1.8)	78 (6.2)		42 (4.0)	211 (11.1)		105 (7.8)	387 (12.9)		
Suffer from any chronic illness or condition										
no	402 (41.1)	307 (24.3)	<0.01	388 (36.7)	421 (22.2)	<0.01	419 (31.3)	569 (19.0)	<0.01	
yes	576 (58.9)	958 (75.7)		670 (63.3)	1479 (77.8)		919 (68.7)	2431 (81.0)		
Limitations in daily activities due to health problems										
not limited at all	492 (50.3)	335 (26.5)	<0.01	727 (68.7)	935 (49.2)	<0.01	525 (39.2)	753 (25.1)	<0.01	
limited but not severely	352 (36.0)	570 (45.1)		234 (22.1)	649 (34.2)		560 (21.9)	1341 (44.7)		
severely limited	134 (13.7)	360 (28.5)		97 (9.2)	316 (16.6)		253 (18.9)	906 (30.2)		
Education										
primary school or less	0	0		285 (26.9)	685 (36.1)	<0.01	205 (15.3)	487 (16.2)	<0.01	
secondary	1589 (82.1)	2310 (87.8)	<0.01	574 (54.3)	1000 (52.6)		923 (68.8)	2170 (72.3)		
high/tertiary	346 (17.9)	320 (12.2)		199 (18.8)	215 (11.3)		214 (15.9)	346 (11.5)		
Number of years spent in paid work arithmetic mean ± standard deviation	34.9 ± 8.5	34.2 ± 9.2	0.042	33.8 ± 10.0	33.7 ± 12.0	0.210	32.5 ± 9.9	32.4 ± 10.0	0.233	
At risk of poverty or social exclusion										
no	1694 (87.5)	2209 (84.0)	0.001	855 (80.8)	1158 (60.9)	<0.01	962 (71.7)	1948 (64.9)	<0.01	
yes	241 (12.5)	421 (16.0)		203 (19.2)	742 (39.1)		380 (28.3)	1055 (35.1)		
HOUSEHOLD LEVEL										
Household size										
one person	336 (17.4)	350 (13.3)	<0.01	167 (15.8)	386 (20.3)	<0.01	389 (29.0)	734 (24.4)	<0.01	
two persons	969 (50.1)	1323 (50.3)		372 (35.2)	607 (31.9)		627 (46.7)	1600 (53.3)		
three or more persons	630 (32.6)	957 (36.4)		519 (49.1)	907 (47.7)		326 (24.3)	669 (22.3)		
Financial burden of total housing costs										
not a burden at all	422 (21.8)	172 (6.5)	<0.01	464 (43.9)	1428 (75.2)	<0.01	182 (13.8)	89 (3.0)	<0.01	
a slight burden	1072 (55.4)	1319 (50.2)		507 (48.0)	452 (23.8)		571 (43.2)	1060 (35.8)		
a heavy burden	441 (22.8)	1139 (43.3)		86 (8.1)	19 (1.0)		570 (43.1)	1812 (61.2)		
Ability to make ends meet										
easily or very easily	423 (21.9)	250 (9.5)	<0.01	49 (4.6)	6 (0.3)	<0.01	101 (7.5)	52 (1.7)	<0.01	
fairly easily or with some difficulty	1185 (61.2)	1614 (61.4)		610 (57.7)	470 (24.7)		754 (56.2)	1364 (45.4)		
with difficulty or with great difficulty	327 (16.9)	766 (29.1)		399 (37.7)	1424 (74.9)		487 (36.3)	1587 (52.8)		
Severely materially deprived household										
not severely deprived	1868 (96.5)	2488 (94.6)	<0.01	981 (92.7)	1440 (75.8)	<0.01	1203 (89.6)	2543 (84.7)	<0.01	
severely deprived	67 (3.5)	142 (5.4)		77 (7.3)	460 (24.2)		139 (10.4)	460 (15.3)		

*n*—number of the respondents; *p*—*p*-value.

**Table 6:** Multivariate logistic regression models with likelihood financial burden of medical care among the older-aged population in Slovenia, Serbia, and, Croatia as an outcome variable.

Participants’ Characteristic	Slovenia		Serbia		Croatia	
	Model 1	Model 2	Model 1	Model 2	Model 1	Model 2
	OR (95% CI)	OR (95% CI)	OR (95% CI)	OR (95% CI)	OR (95% CI)	OR (95% CI)
PERSONAL LEVEL						
Age group						
65–69	1	1	1	1	-	-
70–74	1.05 (0.75–1.47)	1.02 (0.73–1.43)	1.23 (1.00–1.52)	1.24 (1.00–1.54)	-	-
75–79	0.95 (0.66–1.38)	0.93 (0.64–1.35)	1.05 (0.84–1.31)	1.07 (0.86–1.34)	-	-
80+	0.77 (0.51–1.14)	0.78 (0.52–1.18)	0.97 (0.76–1.23)	1.01 (0.79–1.29)	-	-
Marital status						
single/separated/ divorced/widowed	1	1	-	-	1	1
married/cohabit	1.84 (1.35–2.50) *	1.54 (1.03–2.03) *	-	-	1.38 (1.22–1.56) *	1.43 (1.26–1.62) *
Self-perceived general health status						
very good	-	-	1	1	1	1
good	-	-	2.10 (0.85–5.19)	1.71 (0.67–4.35)	0.90 (0.55–1.49)	0.85 (0.51–1.43)
fair	-	-	2.43 (1.00–5.89)	1.91 (0.77–4.77)	1.34 (0.81–2.21)	1.17 (0.70–1.94)
bad	-	-	5.11 (2.08–12.52) *	3.56 (1.41–8.95) *	1.86 (1.11–3.12) *	1.53 (0.90–2.60)
very bad	-	-	8.11 (3.17–20.77) *	5.23 (1.99–13.76) *	2.11 (1.21–3.68) *	1.74 (0.98–3.08)
Suffer from any chronic illness or condition						
no	-	-	1	1	1	1
yes	-	-	1.04 (0.85–1.27)	1.04 (0.85–1.28)	0.84 (0.70–1.00) *	0.84 (0.70–1.00)
Limitations in daily activities due to health problems						
not limited at all	1	1	1	1	1	1
limited but not severely	1.56 (1.15–2.14) *	1.51 (1.10–2.07) *	0.83 (0.68–1.03)	0.82 (0.67–1.02)	1.18 (1.00–1.40)	1.18 (0.99–1.40)
severely limited	2.22 (1.53–3.20) *	2.05 (1.41–2.98) *	1.02 (0.76–1.36)	1.00 (0.74–1.34)	1.05 (0.83–1.32)	1.03 (0.81–1.30)
Education						
primary school or less	-	-	1	1	-	-
secondary	1	1	0.97 (0.81–1.18)	0.95 (0.78–1.16)	-	-
high/tertiary	1.95 (1.42–2.68) *	2.03 (1.47–2.81) *	0.97 (0.74–1.27)	1.03 (0.78–1.36)	-	-
Number of years spent in paid work	1.00 (0.99–1.02)	1.01 (0.99–1.02)	-	-	-	-
At the risk of poverty or social exclusion						
no	1	1	1	1	-	-
yes	0.74 (0.49–1.12)	0.75 (0.48–1.15)	1.57 (1.32–1.87) *	1.03 (0.81–1.32)	-	-
HOUSEHOLD LEVEL						
Household size						
one person		1		-		-
two persons		1.47 (0.93–2.35)		-		-
three and more persons		0.86 (0.50–1.49)		-		-
Financial burden of total housing costs						
not a burden at all		1		1		1
a slight burden		0.98 (0.73–1.32)		0.69 (0.56–0.84) *		1.14 (0.98–1.32)
a heavy burden		0.51 (0.31–0.84) *		0.47 (0.26–0.87) *		0.62 (0.46–0.84) *
Ability to make ends meet						
easily or very easily		-		1		1
fairly easily or with some difficulty		-		3.58 (1.05–12.17) *		2.08 (1.41–3.06) *
with difficulty or with great difficulty		-		6.80 (1.99–23.28) *		2.52 (1.67–3.79) *
Severely materially deprived household						
not severely deprived		-		1		1
severely deprived		-		1.30 (0.98–1.71)		1.05 (0.86–1.27)

OR—odds ratio; CI—confidence interval; * *p* < 0.05.

**Table 7:** Multivariate logistic regression models for the financial burden of dental care.

Participants’ Characteristics	Slovenia		Serbia		Croatia	
	Model 1	Model 2	Model 1	Model 2	Model 1	Model 2
	OR (95% CI)	OR (95% CI)	OR (95% CI)	OR (95% CI)	OR (95% CI)	OR (95% CI)
PERSONAL LEVEL						
Sex						
man	1	1	-	-	-	-
woman	1.02 (0.79–1.31)	1.04 (0.80–1.34)	-	-	-	-
Age group						
65–69	1	1	-	-	-	-
70–74	0.94 (0.71–1.27)	0.94 (0.70–1.27)	-	-	-	-
75–79	0.86 (0.62–1.19)	0.88 (0.63–1.23)	-	-	-	-
80+	0.87 (0.61–1.24)	0.93 (0.65–1.33)	-	-	-	-
Marital status						
single/separated/ divorced/widowed	1	1	-	-	1	1
married/cohabit	1.42 (1.10–1.83) *	1.54 (1.10–2.17) *	-	-	1.25 (1.08–1.46) *	1.18 (0.97–1.45)
Self-perceived general health status						
very good	-	-	1	1	-	-
good	-	-	0.61 (0.26–1.46)	0.50 (0.20–1.26)	-	-
fair	-	-	0.77 (0.34–1.76)	0.61 (0.25–1.48)	-	-
bad	-	-	1.21 (0.52–2.81)	0.86 (0.35–2.11)	-	-
very bad	-	-	1.17 (0.46–2.95)	0.73 (0.27–1.98)	-	-
Suffer from any chronic illness or condition						
no	-	-	1	1	-	-
yes	-	-	1.28 (0.98–1.67)	1.29 (0.98–1.70)	-	-
Limitations in daily activities due to health problems						
not limited at all	-	-	-	-	-	-
limited but not severely	-	-	-	-	-	-
severely limited	-	-	-	-	-	-
Education						
primary school or less	-	-	-	-	1	1
secondary	1	1	-	-	1.11 (0.89–1.39)	1.20 (0.95–1.45)
high/tertiary	1.43 (1.08–1.90) *	1.76 (1.30–2.39) *	-	-	1.33 (1.00–1.75) *	1.56 (1.17–2.10) *
Number of years spent in paid work	1.01 (1.00–1.03)	1.02 (1.00–1.04) *	-	-	-	-
At risk of poverty or social exclusion						
no	-	-	1	1	-	-
yes	-	-	1.51 (1.17–1.95) *	1.03 (0.74–1.44)	-	-
HOUSEHOLD LEVEL						
Household size						
one person		1		-		1
two persons		0.91 (0.62–1.35)		-		1.15(0.90–1.49)
three or more persons		0.99 (0.65–1.51)		-		1.25 (0.97–1.60)
Financial burden of total housing costs						
not a burden at all		1		1		1
a slight burden		0.78 (0.58–1.05)		0.35 (0.27–0.45) *		1.02 (0.86–1.21)
a heavy burden		0.53 (0.34–0.82) *		0.13 (0.06–0.30) *		0.59 (0.42–0.83) *
Ability to make ends meet						
easily or very easily		1		-		1
fairly easily or with some difficulty		1.78 (1.26–2.51) *		-		1.70 (1.10–2.61) *
with difficulty or with great difficulty		1.49 (0.93–2.37)		-		1.81 (1.15–2.86) *
Severely materially deprived household						
not severely deprived		-		1		-
severely deprived		-		1.58 (0.99–2.50)		-

OR—odds ratio; CI—confidence interval; * *p* < 0.05.

**Table 8:** Multivariate logistic regression models for the financial burden of medicines.

Participants’ Characteristics	Slovenia		Serbia		Croatia	
	Model 1	Model 2	Model 1	Model 2	Model 1	Model 2
	OR (95% CI)	OR (95% CI)	OR (95% CI)	OR (95% CI)	OR (95% CI)	OR (95% CI)
PERSONAL LEVEL						
Sex						
man	1	1	-	-	-	-
woman	1.02 (0.79–1.31)	1.04 (0.80–1.34)	-	-	-	-
Age group						
65–69	1	1	-	-	-	-
70–74	0.94 (0.71–1.27)	0.94 (0.70–1.27)	-	-	-	-
75–79	0.86 (0.62–1.19)	0.88 (0.63–1.23)	-	-	-	-
80+	0.87 (0.61–1.24)	0.93 (0.65–1.33)	-	-	-	-
Marital status						
single/separated/ divorced/widowed	1	1	-	-	1	1
married/cohabit	1.42 (1.10–1.83) *	1.54 (1.10–2.17) *	-	-	1.25 (1.08–1.46) *	1.18 (0.97–1.45)
Self-perceived general health status						
very good	-	-	1	1	-	-
good	-	-	0.61 (0.26–1.46)	0.50 (0.20–1.26)	-	-
fair	-	-	0.77 (0.34–1.76)	0.61 (0.25–1.48)	-	-
bad	-	-	1.21 (0.52–2.81)	0.86 (0.35–2.11)	-	-
very bad	-	-	1.17 (0.46–2.95)	0.73 (0.27–1.98)	-	-
Suffer from any chronic illness or condition						
no	-	-	1	1	-	-
yes	-	-	1.28 (0.98–1.67)	1.29 (0.98–1.70)	-	-
Limitations in daily activities due to health problems						
not limited at all	-	-	-	-	-	-
limited but not severely	-	-	-	-	-	-
severely limited	-	-	-	-	-	-
Education						
primary school or less	-	-	-	-	1	1
secondary	1	1	-	-	1.11 (0.89–1.39)	1.20 (0.95–1.45)
high/tertiary	1.43 (1.08–1.90) *	1.76 (1.30–2.39) *	-	-	1.33 (1.00–1.75) *	1.56 (1.17–2.10) *
Number of years spent in paid work	1.01 (1.00–1.03)	1.02 (1.00–1.04) *	-	-	-	-
At risk of poverty or social exclusion						
no	-	-	1	1	-	-
yes	-	-	1.51 (1.17–1.95) *	1.03 (0.74–1.44)	-	-
HOUSEHOLD LEVEL						
Household size						
one person		1		-		1
two persons		0.91 (0.62–1.35)		-		1.15 (0.90–1.49)
three or more persons		0.99 (0.65–1.51)		-		1.25 (0.97–1.60)
Financial burden of total housing costs						
not a burden at all		1		1		1
a slight burden		0.78 (0.58–1.05)		0.35 (0.27–0.45) *		1.02 (0.86–1.21)
a heavy burden		0.53 (0.34–0.82) *		0.13 (0.06–0.30) *		0.59 (0.42–0.83) *
Ability to make ends meet						
easily or very easily		1		-		1
fairly easily or with some difficulty		1.78 (1.26–2.51) *		-		1.70 (1.10–2.61) *
with difficulty or with great difficulty		1.49 (0.93–2.37)		-		1.81 (1.15–2.86) *
Severely materially deprived household						
not severely deprived		-		1		-
severely deprived		-		1.58 (0.99–2.50)		-

OR—odds ratio; CI—confidence interval; * *p* < 0.05.

## Data Availability

For using EU-SILC microdata for this research, we have obtained permission from European Commission (Ref. Ares(2019)6720595). Microdata available from Eurostat, on request. Details are available at https://ec.europa.eu/eurostat/web/microdata/european-union-statistics-on-income-and-living-conditions (accessed on 8 March 2022).
